# Microbes and microbiomes in 2020 and beyond

**DOI:** 10.1038/s41467-020-18850-6

**Published:** 2020-10-05

**Authors:** Aravind Natarajan, Ami S. Bhatt

**Affiliations:** 1 Department of Medicine (Hematology, Blood and Marrow Transplantation), Stanford University, Stanford, CA 94305 USA; 2 Department of Genetics, Stanford University, Stanford, CA 94305 USA

**Keywords:** Metagenomics, Microbiome

## Abstract

In the next decade, advances in our understanding of microbes and microbiomes will likely transform our way of life; providing novel therapeutics, alternate energy sources, and shaping fundamental doctrines of biology. We explore the promises herein, and tools required to achieve this progress. Notably, it is critical that we improve the inclusivity and diversity of our research agendas and teams, so that science benefits people of all identities and backgrounds.

Microbes have shaped the course of humanity, enabling basic biological discoveries such as the triplet nature of codons, yielding therapeutics including numerous antibiotics, and contributing to everyday life by serving us fermented products and as sources of enzymes for our laundry detergent. Recent studies have also revealed that our healthy existence is intricately reliant on microbes that inhabit our body and influence physiological functions in ways that we are only beginning to understand. Notably, the entirety of our knowledge of and from microbes is derived from 0.001% of microbial taxa predicted to exist on earth^1^. With advances in technology that enable us to investigate microbes across time and space, humanity has the opportunity ‘*to explore strange new worlds. To seek out new life and new civilizations. To boldly go where no one has gone before!’* guided by reams of sequence information. In this commentary, we reflect on the impact of the burgeoning knowledge of microbes and microbiomes, and some of the tools required to explore this new world.

## Human microbiome

The study of the human microbiome takes us back to the origins of microbiology when Antonie van Leeuwenhoek invented the simple microscope and first saw bacteria (which he termed *animalcules*) derived from pond scum and between his teeth. Through concerted efforts over the last two decades, we now better understand the identity of microbes that inhabit the human body. In certain cases, we even understand the roles of specific microbes in the maintenance of our health. Despite some promising advances, the holy grail of microbe-based therapeutics has under-delivered, perhaps because of premature hype from associative knowledge and absence of causative information. However, as we move past the first wave of exploratory enthusiasm, the field is making rapid progress, developing computational algorithms, genetic tools for microbial manipulation, improved metrics for measurements, and high-throughput experiments to nail down molecular and biochemical details of the complex relationship between human hosts and microbes. We anticipate that understanding the intricate signaling between microbes, and with the host^2,3^ through small molecules and peptides will be a key area of progress that will yield therapeutics and clinical interventions. Further, sequencing technologies and bioinformatic algorithms that are honed to characterize phages, protists and other eukaryotic symbionts of the human body are promising areas of development. Despite the wave of new information that is being generated, one area of concern in studies on the human microbiome is the lack of diversity in research subjects, where most studies focus on western and Caucasian populations. We need to urgently act to ensure that existing disparities in healthcare are not exacerbated as we make forays into new therapeutic avenues backed by our knowledge of the human microbiome^4^.

## Environmental microbiome

In the past decade, we have witnessed fascinating advances in the study of microbes from diverse ecologies, including intricate symbionts at submarine volcanic eruptions^5^, hardy survivors in the Atacama desert (ecology akin to Mars)^6^, and ‘huge phages’ found in wide-ranging ecosystems^7^. Focused efforts like the Tara Expeditions and Earth Microbiome project^8^ that leverage massive sampling in combination with high-throughput sequencing have significantly expanded the catalog of known microbes. While these endeavors are invaluable in advancing our knowledge, it is humbling to remember that despite our best efforts, we are still only sampling a miniscule fraction of the 10^12^ microbial species predicted to currently exist^1^, and an even smaller fraction of the organisms that have ever existed and will exist in the future. In the new decade, we anticipate learning new facets of the rules of life and biocatalyzed chemistries from these novel microorganisms. For instance, strategies to assemble complete genomes^9–11^, track mobile genetic elements^12^ and extrachromosomal elements^13^ may finally allow us to comprehensively grasp the dynamics of the evolution and exchange of genetic material. This will also provide us insight into novel classes of enzymes that interact with nucleic acids in fundamental processes such as DNA repair, epigenetic modifications, and recombination, and have translational utility in genome engineering, just as CRISPR/Cas systems have revolutionized molecular biology. While modern tools and techniques have allowed us to explore new organisms, we are also improving our understanding of well-studied model organisms like *Escherichia coli*. For instance, advances in biochemical, computational and microscopy-based techniques have enabled us to dissect the nature and extent of subcellular organization in this familiar microbe, yielding exciting new fundamental insights.

## Transformative tools and technology

The biggest challenge in honing tools to explore the unknown remains our deep familiarity with the known. Therefore, we need to constantly remember that our world view of microbes and their potential is based on a fraction of the existent diversity of life. One strategy to develop enabling futuristic tools is to remain unfettered by current biological principles, and lean on the fundamental sciences of mathematics, physics and chemistry, and contemporaneous machine learning algorithms. Mathematical frameworks that can handle the inherent nonlinearity, stochasticity and complexity of biological problems, assembling multi-omics data at a systems level, are a cornerstone for progress. Similarly, defining statistical measures that are most relevant to assessing ecological interactions is also invaluable to capture signal over noisy data with reasonable sensitivity. Cutting edge physics has pushed the boundaries of microscopy, down to even visualizing folded proteins^14^ and RNAs^15^. In the next decade, we anticipate non-invasive methods to capture host-microbe and other systems level interactions in vivo. An important element herein is advancements in robust fluorescence markers and strategies in bioorthogonal chemistry that can identify and label different biomolecules such as nucleic acids, cell-surface proteins, and sugars. Peering within a cell, improvements in mass spectrometry, pushing the boundaries of physicochemical principles, have enabled researchers to gain metabolic insights that were previously inaccessible. We hope that such high-throughput strategies in analytical chemistry continue to advance, enabling identification of small molecules ab initio from complex mixtures. These efforts in metabolomics will benefit from innovative bioinformatic algorithms that identify and predict functional pathways in microbes. Similar computational efforts to map the metabolic potential and growth requirements of traditionally unculturable microbes will augment our ability to grow these exotic bugs and understand their biology. Finally, computer algorithms have exponentially transformed the scale of biological investigations, ranging from massive efforts to identify novel microbes, to characterizing molecular pathways, and defining fundamental principles of biochemical and molecular interactions. Further, maturation of machine learning strategies will enable us to take a fresh approach to data analysis, uncluttered by limitations of the human imagination.

## Conclusion

Taken together, in the centuries since the time of van Leeuwenhoek, breakthrough discoveries have exemplified the transformative power of discovering new microbes, understanding their biology, and gaining access to their evolutionarily honed biocatalytic potential. Recognizing that this is just a tiny fraction of the abounding knowledge and resources that exist around us inspires curiosity and verve that in turn fosters endeavors in research. These efforts will certainly improve the quality of our lives and likely even sustain our survival as a species. The steady rise in multi-drug resistant superbugs and extended dry spell in the discovery of novel antibiotics is a major cause for concern. We hope that microbes will continue to yield novel antibiotics and inspire new synthetic solutions. Further, as we make progress in deciphering  the mechanisms that commensal microbes use to influence its human host, we are certain to see a new wave of microbe-based therapeutics, including the potential use of bugs or “bug-derived products” as drugs. The promising impacts of new frontiers in microbiome research extend well beyond medicine. This has never been more evident as we rely on microbial cellulases for the production of biofuels, and realize that microbes are the largest players in the global carbon cycle with a tangible impact on global warming and the health of our planet. At a more fundamental level, we are innately curious about how the world around us works and the delightfully different manifestations of life. While it is certain that the next decade of microbiome research will reveal novel strategies in survival, unimagined metabolic pathways and intricate mechanisms in genetic regulation, it may well challenge more sacrosanct principles such as the central dogma. For instance, microbes did present us nonribosomal peptide synthesis as an alternate route to protein expression.

Finally, as we look ahead and chart the course for our exciting explorations of microbes and microbiomes, it is imperative to take stock of who is privileged to participate in this journey and who reaps the most benefits from its bounties. Scientists of underrepresented identities continue to face systemic challenges that preclude them from fully engaging in research. Further, therapeutics by and large are neither developed for nor tested in diverse populations. The racial, ethnic and socioeconomic inequality in subjects of human microbiome research threatens to perpetuate this disparity in healthcare. While it is beyond the scope of this commentary to deeply assess this problem or to offer solutions, we believe that as we look to the future, it is imperative not only to focus on what we see through the microscope but also to be keenly aware of the context of our work in the broader society.
